# Importance of Fc Receptor γ-Chain ITAM Tyrosines in Neutrophil Activation and *in vivo* Autoimmune Arthritis

**DOI:** 10.3389/fimmu.2019.00252

**Published:** 2019-02-25

**Authors:** Tamás Németh, Krisztina Futosi, Marcell Szabó, Petra Aradi, Takashi Saito, Attila Mócsai, Zoltán Jakus

**Affiliations:** ^1^Department of Physiology, Semmelweis University School of Medicine, Budapest, Hungary; ^2^MTA-SE “Lendület” Lymphatic Physiology Research Group of the Hungarian Academy of Sciences and the Semmelweis University, Budapest, Hungary; ^3^Laboratory for Cell Signaling, RIKEN Center for Integrative Medical Sciences, Yokohama, Japan

**Keywords:** neutrophils, Fc receptor γ-chain, ITAM tyrosines, autoimmune arthritis, Fc receptors

## Abstract

Activating Fcγ receptors associated with Fc receptor γ-chain (FcRγ) are critical for mediating neutrophil effector functions in immune complex-mediated autoimmune diseases. FcRγ contains ITAM tyrosines and the *in vivo* role of these tyrosines has not been defined in neutrophils and arthritis. In this study, the *in vivo* functions of FcRγ ITAM tyrosines were characterized using wild type and ITAM tyrosine mutant (Y65F/Y76F) transgenic mice crossed to an FcRγ-deficient genetic background. FcRγ-deficient neutrophils showed undetectable cell surface expression of the activating Fcγ receptor IV, defective immune complex-induced superoxide production, degranulation and spreading. Although the re-expression of both the wild type and the ITAM tyrosine mutant (Y65F/Y76F) FcRγ could restore activating Fcγ receptor expression of FcRγ-deficient neutrophils, only the wild type transgenic form could mediate Fcγ receptor-dependent effector functions. In contrast, neutrophils carrying ITAM tyrosine mutant FcRγ were unable to produce superoxide, mediate degranulation and perform active spreading. In addition, our results confirmed the protection of FcRγ-deficient mice from autoimmune arthritis. Importantly, the presence of the wild type FcRγ transgene, in contrast to the ITAM tyrosine mutant transgene, partially reversed autoimmune arthritis development. The reversing effect of the wild type transgene was even more robust when animals carried the wild type transgene in a homozygous form. Collectively, FcRγ ITAM tyrosines play a critical role in the induction of neutrophil effector responses, the initiation and progression of an autoantibody-induced experimental arthritis *in vivo*, indicating a signaling, rather than just a receptor stabilizing function of the molecule.

## Introduction

Activating Fcγ receptors expressed on the surface of neutrophils are critical for mediating various cellular responses including immune complex-triggered cellular activation, clearance of immune complexes and phagocytosis of opsonized particles (1–3). These processes are involved in the pathogenesis of various autoimmune diseases including rheumatoid arthritis (2–4).

Resting human neutrophils express the activating Fcγ receptors FcγRIIA and FcγRIIIB, while FcγRI appears upon stimulation (5). While the single chain transmembrane FcγRIIA contains an immunoreceptor tyrosine-based activation motif (ITAM) in the cytoplasmic tail of the molecule, FcγRI is associated with an ITAM-bearing transmembrane adapter protein called Fc receptor γ-chain (FcRγ). In addition, the GPI-anchored FcγRIIIB is also present on the surface of human neutrophils. This molecule does not contain an ITAM-motif and is not associated with an ITAM-bearing adapter side chain; according to the current view it is functionally connected to FcγRIIA (5, 6).

Murine neutrophils express a different set of activating Fc receptors including two low affinity (FcγRIII and FcγRIV) and one high affinity (FcγRI) Fcγ receptors, all of which are associated with the ITAM-containing FcRγ (5). Our previous findings indicated the critical but overlapping role of FcγRIII and FcγRIV in immune complex-dependent neutrophil activation (6). Subsequent *in vivo* studies revealed the importance of FcγRIII and FcγRIV for the development and progression of autoantibody-induced arthritis and autoimmune valvular carditis in the K/BxN serum transfer experimental model (7, 8).

As discussed above, all activating murine Fcγ receptors form a complex with FcRγ, which molecule does not contain a ligand binding domain (1). It is known that the lack of FcRγ abrogates the cell surface expression of activating Fcγ receptors and FcRγ-deficiency leads to abolished Fc receptor-dependent neutrophil effector responses and protection from autoimmune arthritis (6, 9–13). However, due to the absence of the cell surface expression of activating Fcγ receptors in FcRγ-deficient mice, it remains unclear whether the sole function of FcRγ is to enable the receptor expression or it is also actively involved in the signaling process through its ITAM tyrosines.

In prior *in vitro* structure-function studies, the role of ITAM tyrosine phosphorylation was demonstrated in serotonin secretion in a basophilic cell line suggesting the signaling function of FcRγ ITAM tyrosines (14). It was also reported that the phosphorylation of the ITAM tyrosines is induced by the FcRγ-associated FcεR stimulation in mast cells (15).

The functional role of these ITAM tyrosines was characterized using FcRγ-deficient mice reconstituted with murine wild type and ITAM tyrosine mutant (Y65F/Y76F) transgenes. These findings suggested that the ITAM tyrosines are involved in degranulation, cytokine production, prostaglandin synthesis and passive systemic anaphylaxis in mast cells (16). In another genetic model for *in vivo* studies, human transgenic FcRγ was expressed carrying mutated ITAM tyrosines on an FcRγ-deficient genetic background (NOTAM mice) (17). While the surface expression of Fc receptors was not affected, the *in vivo* cytotoxicity critically depended on FcRγ ITAM signaling (17). The uptake of immune complexes and the cross presentation of antigens was reported to be regulated by FcRγ ITAM signaling in dendritic cells, while MHC class II antigen presentation was ITAM-independent (18). In contrast to the first two reports suggesting the functions of FcRγ ITAM tyrosines, recent mouse studies revealed that daratumumab, which is a monoclonal therapeutic antibody targeting CD38 that is highly expressed on the surface of some kinds of tumor cells, induces cancer cell death after its binding, which process occurs in NOTAM but not in FcRγ-deficient mice after blocking FcγRIIB (19). In addition, Lehmann et al. showed that engineered chimeric antibodies instructed splenic dendritic cells to activate CD4- and CD8-positive T-cells through the FcRγ-coupled FcγRIV without the involvement of the ITAM tyrosines (20). Collectively, these recent reports indicated the existence of ITAM-independent *in vivo* functions of FcRγ-coupled activating Fc receptors (19, 20). Therefore, further studies are needed to define the *in vivo* role of FcRγ ITAM tyrosines.

Upon Fc receptor-stimulation of neutrophils, FcRγ was reported to be phosphorylated and to recruit the Syk tyrosine kinase, which promotes activation of the distal signaling pathways and induces cellular effector responses (6, 21, 22). However, the functional role of the FcRγ ITAM tyrosines has not been directly tested in neutrophils and neutrophil-dependent autoimmune diseases *in vivo*. Herein, we used a genetic approach to characterize the possible role of FcRγ ITAM tyrosines in neutrophils and *in vivo* autoimmune arthritis. We demonstrated that FcRγ ITAM tyrosines are required for the immune complex-dependent activation of neutrophils and the development and progression of experimental autoimmune arthritis.

## Materials and Methods

### Animals

FcRγ-deficient (*Fcer1g*^tm1Rav/tm1Rav^, referred to as FcRγ KO) mice were purchased from Taconic Farms (Hudson, NY, USA) (10). Animals expressing the wild type and the ITAM tyrosine mutant FcRγ (where tyrosines at positions 65 and 76 were replaced by phenylalanines) were described previously and were crossed with FcRγ KO mice (referred to as FcRγ KO + WT FcRγ Tg and FcRγ KO + YF FcRγ Tg animals, respectively) (16). To augment the expression of the transgenic wild type and mutant FcRγ chain, the mice were crossed to obtain homozygous, double transgenic animals (referred to as FcRγ KO + 2x WT FcRγ Tg and FcRγ KO + 2x YF FcRγ Tg mice, respectively). Single and double transgenic animals were differentiated by quantitative PCR. Mice carrying the KRN T-cell-receptor transgene were maintained in heterozygous form by mating with C57BL/6 mice (23). All transgenic mice were backcrossed to the C57BL/6 genetic background. Genotyping was performed by allele-specific PCR.

Wild type control C57BL/6 mice were purchased from Charles River or the Hungarian National Institute of Oncology (Budapest, Hungary). NOD mice, as well as a congenic strain carrying the CD45.1 allele on the C57BL/6 genetic background (B6.SJL-*Ptprc*^*a*^) were purchased from the Jackson Laboratory.

Mice were kept in individually ventilated cages (Tecniplast) in a conventional facility. All animal experiments were approved by the Animal Experimentation Review Board of the Semmelweis University.

Bone marrow chimeras were generated by intravenous injection of unfractionated bone marrow cells into recipients carrying the CD45.1 allele on the C57BL/6 genetic background, which were lethally irradiated before by 11 Gy from a ^137^Cs source using a Gamma-Service Medical (Leipzig, Germany) D1 irradiator. 4 weeks after transplantation, peripheral blood samples were stained for Ly6G and CD45.2 (Clones 1A8 and 104, respectively; both from BD Biosciences) and analyzed by a BD Biosciences FACSCalibur flow cytometer as previously described (21).

### K/B × N Serum-Transfer Arthritis

Mice carrying the KRN T-cell receptor transgene on the C57BL/6 genetic background were mated with NOD mice to obtain transgene-positive (arthritic) K/B × N and transgene-negative (control) B × N mice (23, 24). The presence of the transgene was determined by allele-specific PCR and confirmed by phenotypic assessment. Blood was taken by retroorbital bleeding and sera from arthritic and control mice were pooled separately.

Arthritis was induced by a single intraperitoneal injection of 400 μl K/B × N (arthritic) or B × N (control) serum into intact mice or bone marrow chimeras, followed by daily assessment of arthritis severity for 2 weeks as described (24–26). Visible clinical signs were scored on a 0–10 scale by two investigators blinded for the origin and treatment of the mice. Ankle thickness was measured by a spring-loaded caliper (Kroeplin). For histological analysis, mice were sacrificed on Day 8, their fore limbs were fixed in 4% paraformaldehyde, decalcified (Osteomoll, Merck Millipore) dehydrated, embedded in paraffin, sectioned at 9 μm thickness and stained with hematoxylin and eosin.

### Isolation and Activation of Neutrophils

Mouse neutrophils were isolated from the bone marrow of the femurs and tibias of intact mice or chimeras by hypotonic lysis followed by Percoll (GE Healthcare) gradient centrifugation using sterile and endotoxin-free reagents as described (25–27). Cell surface FcγRIV expression was detected by an anti-FcγRIV antibody (Clone 9E9; a gift from Prof. Jeffrey V. Ravetch). A secondary staining with FITC labeled anti-Armenian and Syrian hamster antibodies (Clones G70-204 and G94-56; BD Biosciences) was performed.

Neutrophils were kept at room temperature in Ca^2+^- and Mg^2+^-free medium until use and prewarmed to 37°C prior to activation. Neutrophil assays were performed at 37°C in HBSS supplemented with 20 mM HEPES, pH 7.4. To obtain immobilized immune complex-coated surfaces, human lactoferrin (LFR; Sigma-Aldrich) was either bound directly to regular Nunc MaxiSorp F96 (Thermo Fisher) plates (for superoxide release and degranulation measurements) or covalently linked to poly-L-lysine-coated 24-well tissue culture plates (for spreading assays) and then treated with rabbit anti-LFR IgG (Sigma-Aldrich) as described (6). Superoxide release by neutrophils was followed by a cytochrome c reduction test from 100 μl aliquots of 4 × 10^6^/ml cells plated on immobilized immune complexes or on 10% fetal cow serum (FCS) in the presence of PMA as described (25). The release of gelatinase was determined by gelatinase zymography as previously described (6). Spreading of the cells was followed by phase contrast microscopy.

### Biochemical Studies

For analysis of protein contents, neutrophils were lysed in 100 mM NaCl, 30 mM Na-HEPES (pH 7.4), 20 mM NaF, 1 mM Na-EGTA, 1% Triton X-100, 1 mM benzamidine, freshly supplemented with 0.1 U/ml Aprotinin, 1:100 Mammalian Protease Inhibitor Cocktail, 1:100 Phosphatase Inhibitor Cocktail 2, 1 mM PMSF and 1 mM Na_3_VO_4_ (all from Sigma-Aldrich). After removal of insoluble material, lysates were boiled in sample buffer. Whole cell lysates were run on SDS-PAGE and immunoblotted using antibodies against FcRγ (Host: rabbit; Upstate) or β-actin (Clone AC-74; Sigma-Aldrich) and by peroxidase-labeled secondary antibodies (GE Healthcare). The signal was developed using the ECL system (GE Healthcare) and exposed to X-ray films.

### Presentation of the Data and Statistical Analysis

Experiments were performed the indicated number of times. Quantitative graphs and kinetic curves show mean and SEM from all independent *in vitro* experiments or from all individual mice from the indicated number of experiments. Statistical analyses were carried out by the STATISTICA software using two-way (factorial) ANOVA, with treatment and genotype being the two independent variables. In case of kinetic assays, area under the curve (AUC) was used for statistical analysis. *P* values below 0.05 were considered statistically significant.

## Results

### FcRγ-Deficient Neutrophils Have Impaired Immune Complex-Mediated Cell Responses and Lack Activating Fc Receptors From the Cell Surface

**Figure 1:**
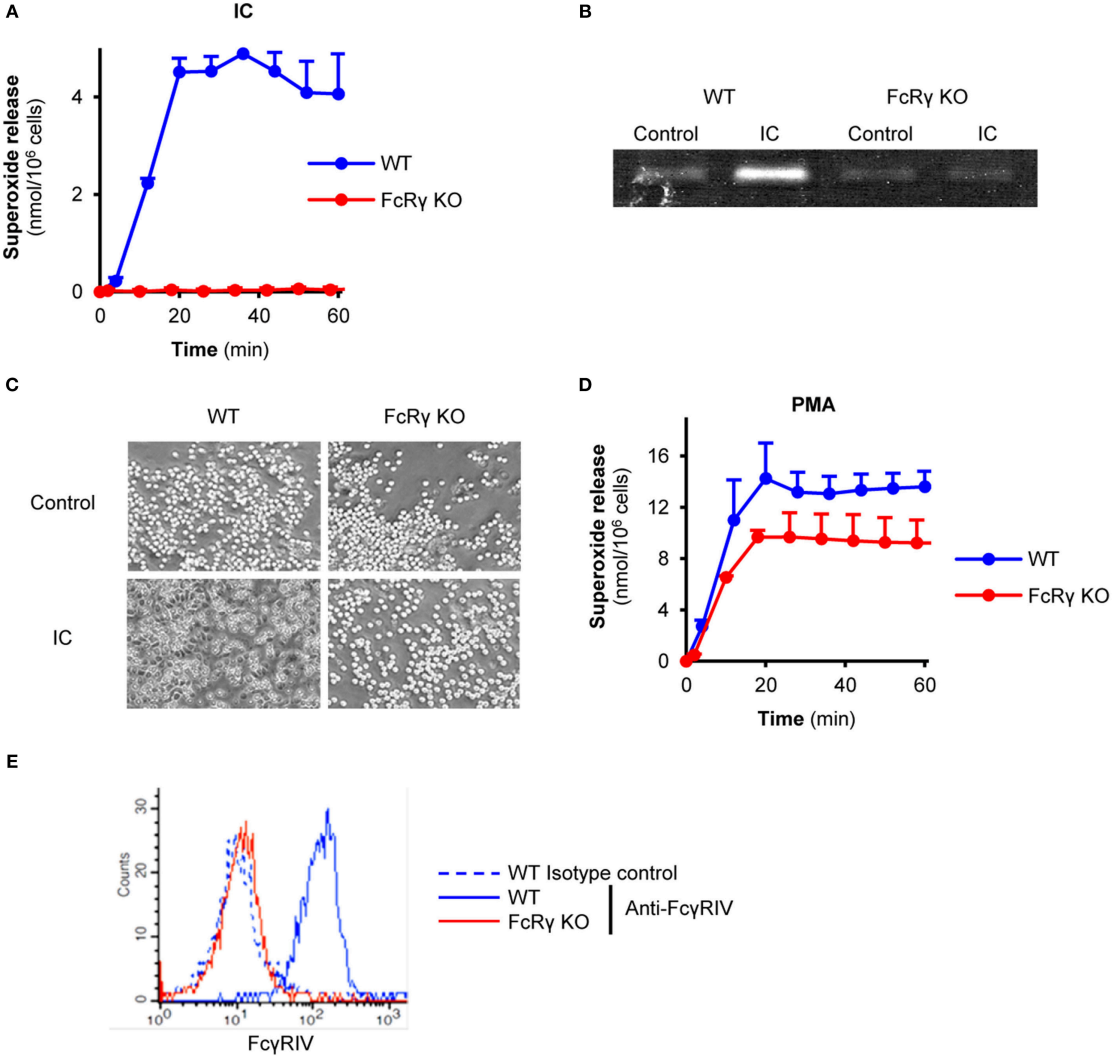
Impaired effector responses and activating Fc receptor-expression in FcRγ-deficient neutrophils. Wild type (WT) and FcRγ-deficient (FcRγ KO) neutrophils were plated on immobilized immune complexes and their superoxide release **(A)**, gelatinase degranulation **(B)**, and spreading **(C)** was followed. The ability of FcRγ KO cells to produce superoxide anions was tested by PMA stimulation (at a 100 nM concentration) **(D)**. Neutrophil cell surface FcγRIV expression was detected by flow cytometry **(E)**. The gelatinase zymogram, the photos and the flow cytometric curves are representative of 3–7 independent experiments. Kinetic curves in **(A,D)** show mean and SEM of 3 independent experiments. Control data points were subtracted. See the text for actual *P* values. IC, Immune complex; PMA, Phorbol myristate acetate.

First, we characterized the phenotype of the FcRγ-deficient neutrophils. When plating on immobilized immune complex surfaces, freshly isolated wild type mouse neutrophils were able to produce superoxide, while in the absence of the FcRγ the cell response was abolished (Figure 1A; *P* = 1.3 × 10^−4^). In line with this finding, FcRγ-deficient neutrophils were unable to release the granule marker gelatinase or perform cell spreading (Figures 1B,C; *P* = 2.6 × 10^−4^ and *P* = 1.4 × 10^−4^). Meanwhile, the absence of the adaptor chain did not affect Fc receptor-independent superoxide production triggered by PMA compared to wild type cells (Figure 1D; *P* = 0.81). However, the activating, FcRγ-associated Fcγ receptor IV was found to be absent from the cell surface of FcRγ-deficient neutrophils (Figure 1E; *P* = 4.4 × 10^−5^).

### Restoration of FcRγ-Expression in Neutrophils by Wild Type and ITAM Mutant FcRγ

**Figure 2:**
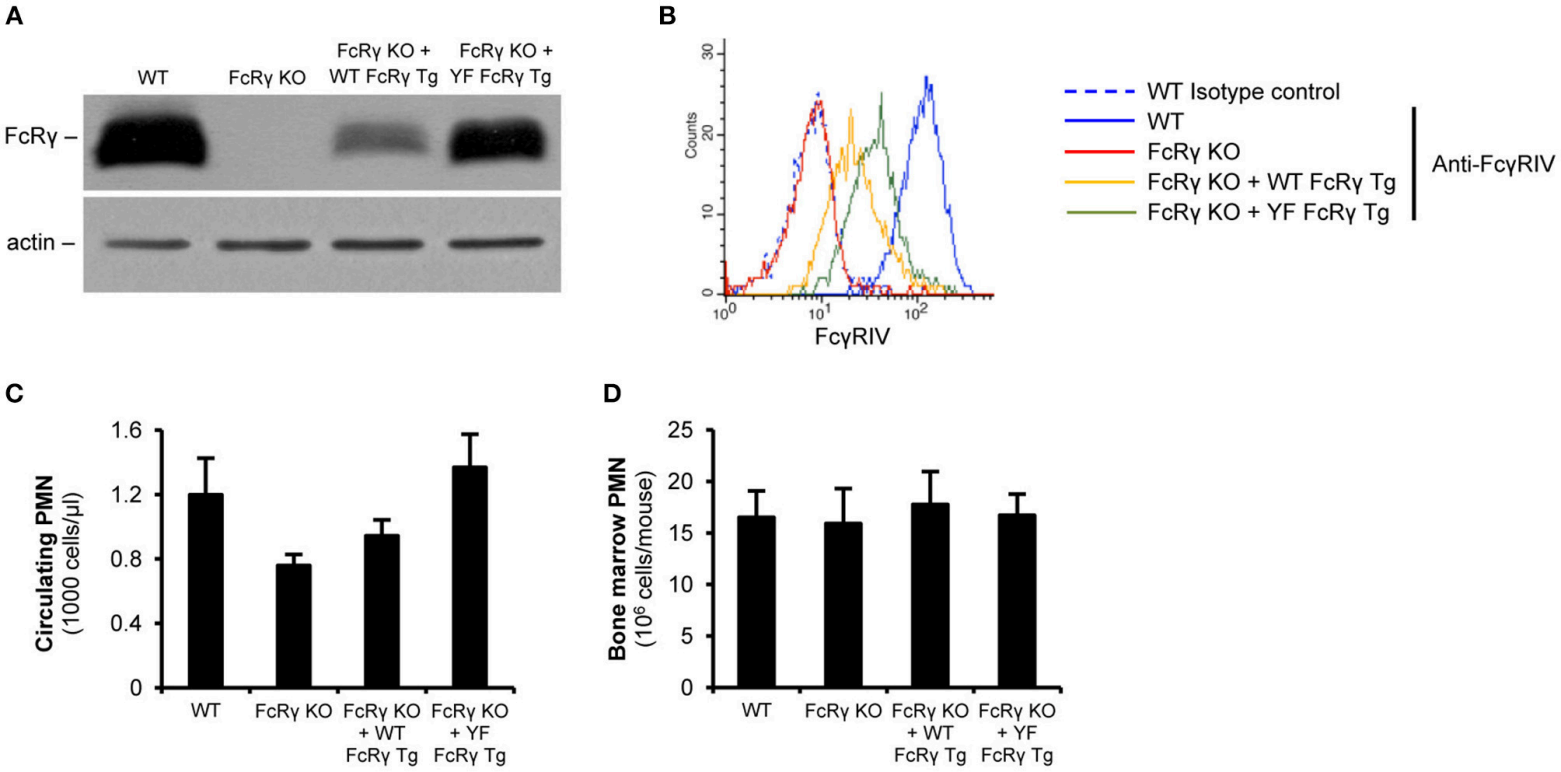
Restoration of (cell surface) expression of FcRγ in neutrophils by wild type and ITAM mutant FcRγ transgenes on an FcRγ-deficient background. FcRγ expression was tested by immunoblotting on whole cell lysates of wild type (WT), FcRγ-deficient (FcRγ KO), wild type FcRγ transgenic (FcRγ KO + WT FcRγ Tg) or ITAM tyrosine mutant FcRγ transgenic (FcRγ KO + YF FcRγ Tg) neutrophils **(A)**. Cell surface expression of the activating receptor FcγRIV was followed by flow cytometry **(B)**. The blot and the flow cytometric curves are representative of 6–7 independent experiments. The circulating neutrophil (PMN) counts are presented in wild type (WT), FcRγ-deficient (FcRγ KO), wild type FcRγ transgenic (FcRγ KO + WT FcRγ Tg) or ITAM tyrosine mutant FcRγ (FcRγ KO + YF FcRγ Tg) neutrophils **(C)**. The average bone marrow-isolated neutrophil numbers did not differ significantly in the different genotypes **(D)**. **(C,D)** Show mean and SEM from 2 to 11 independent experiments. See the text for actual *P* values. PMN, Polymorphonuclear cells (neutrophils).

For investigating the role of the intracellular ITAM tyrosines in various cell responses, we re-expressed wild type or ITAM tyrosine mutant FcRγ in mice on an FcRγ-deficient background. According to the immunoblot results, neutrophils from the wild type and the ITAM tyrosine mutant FcRγ transgenic mice could produce the protein (Figure 2A; the expression level was 24% and 68% of the original wild type level, respectively; *P* = 2.2 × 10^−3^ for FcRγ KO + WT FcRγ Tg vs. FcRγ KO). As a consequence, the activating Fcγ receptor IV appeared on the cell surface of the two different transgenic neutrophil populations (Figure 2B; the expression level was 25 and 43% of the maximum, respectively; *P* = 0.026 for FcRγ KO + WT FcRγ Tg vs. FcRγ KO). We further analyzed the neutrophil counts in the periphery or in the bone marrow in wild type, FcRγ-deficient, wild type and ITAM mutant FcRγ-expressing mice and we did not detect significant differences in the sizes of the granulocyte populations (Figures 2C,D; *P* = 0.09 and *P* = 0.97, respectively). This indicated that transgenic re-expression of wild type and ITAM tyrosine mutant FcRγ did not interfere with neutrophil survival and viability.

### FcRγ ITAM Tyrosines Are Critical for Immune Complex-Mediated Neutrophil Cell Responses

**Figure 3:**
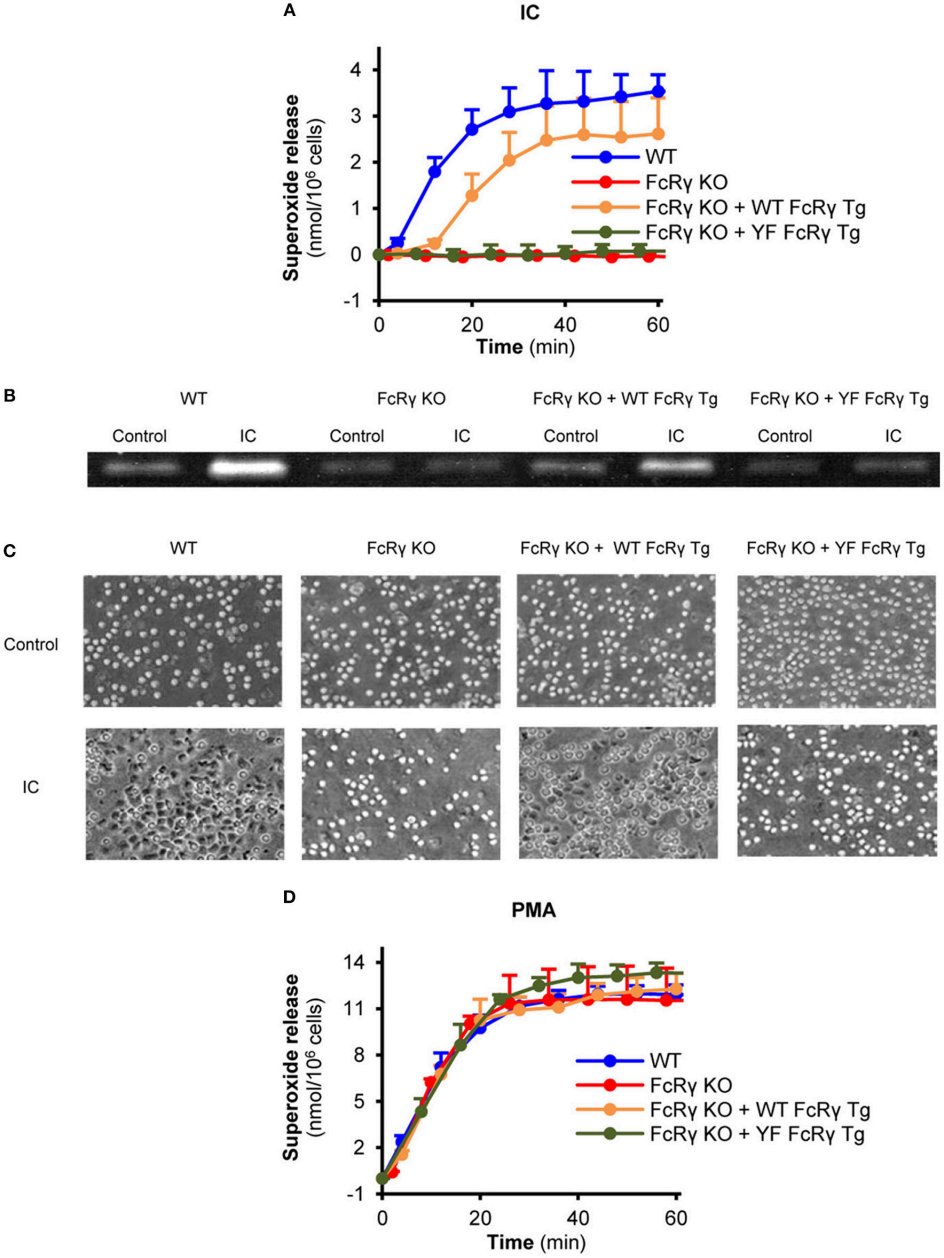
Critical role of the intracellular FcRγ ITAM tyrosines in mediating neutrophil cell responses *in vitro*. Wild type (WT), FcRγ-deficient (FcRγ KO), wild type FcRγ transgenic (FcRγ KO + WT FcRγ Tg) or ITAM tyrosine mutant FcRγ transgenic (FcRγ KO + YF FcRγ Tg) neutrophils were plated on immobilized immune complex surfaces and their superoxide release **(A)**, gelatinase release **(B)** and cell spreading **(C)** was detected. The functionality of all genotypes was tested by PMA stimulation **(D)**. Kinetic curves in **(A,D)** represent mean and SEM from 3 independent experiments. The gelatinase zymogram and the photos are representative of 3 independent experiments. See the text for actual *P* values. IC, Immune complex; PMA, Phorbol myristate acetate.

Compared to FcRγ-deficient neutrophils, re-expression of the wild type FcRγ could partially restore the Fc receptor-mediated release of superoxide, while this response was totally blocked in connection with ITAM tyrosine mutant FcRγ-bearing cells (Figure 3A; *P* = 0.017 for FcRγ KO + WT FcRγ Tg vs. FcRγ KO + YF FcRγ Tg and *P* = 0.75 for FcRγ KO + YF FcRγ Tg vs. FcRγ KO). The same essential role of these tyrosines could be observed in connection with gelatinase degranulation and cell spreading (Figures 3B,C; *P* = 0.04 for FcRγ KO + WT FcRγ Tg vs. FcRγ KO + YF FcRγ Tg and *P* = 0.02 for FcRγ KO + WT FcRγ Tg vs. FcRγ KO + YF FcRγ Tg, respectively). In contrast to immune complex-triggered conditions, PMA induced a robust superoxide production in all genotypes (Figure 3D; *P* = 0.90).

### Essential Role of FcRγ ITAM Tyrosines in Arthritis Development

**Figure 4:**
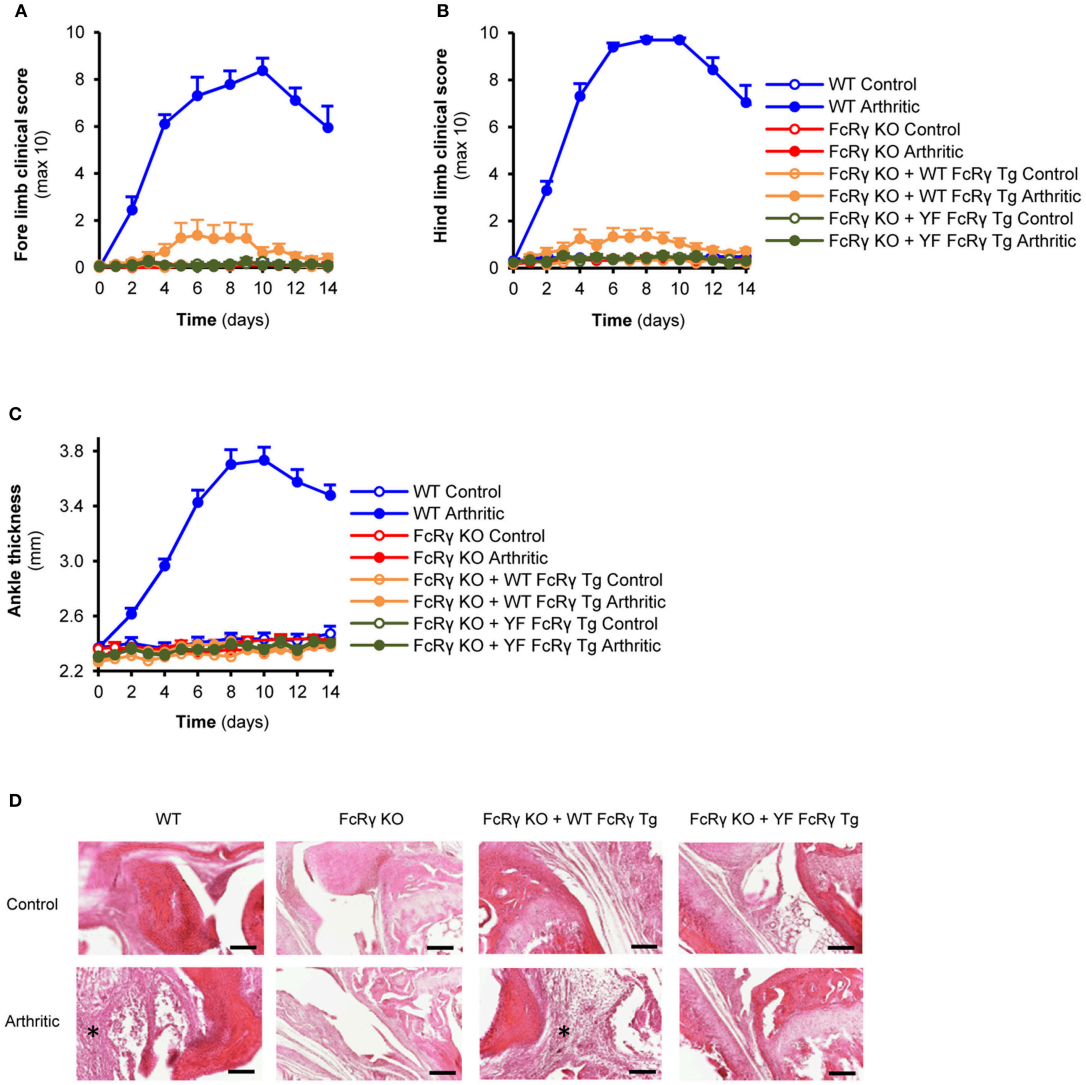
Re-expression of the wild type but not the ITAM tyrosine mutant FcRγ transgene partially reverses the arthritic phenotype. Wild type (WT), FcRγ-deficient (FcRγ KO), wild type FcRγ transgenic (FcRγ KO + WT FcRγ Tg) or ITAM tyrosine mutant FcRγ transgenic (FcRγ KO + YF FcRγ Tg) animals were injected with B × N (Control) or K/B × N (Arthritic) serum intraperitonally on Day 0. Arthritis development was followed by clinical scoring of the fore limbs **(A)** and the hind limbs **(B)** and ankle thickness measurement **(C)**. Leukocyte infiltration was visualized by microscopy after hematoxylin and eosin staining [**(D)**, 20x original magnification, bars: 100 μm; asterisks point at infiltrating leukocytes]. Quantitative data show mean and SEM from 6 to 7 control and 6 to 7 arthritic serum-treated individual mice per group from 3 independent experiments **(A–C)**. See the text for actual *P* values.

For the *in vivo* studies, besides using intact mice, we also generated bone marrow, radiation chimeras in order to investigate the role of the FcRγ ITAM tyrosines in the myeloid compartment. In general, we did not see substantial differences between the arthritic phenotypes of the intact animals and the bone marrow chimeras. When receiving K/BxN serum, a small, but significant visible inflammation appeared on the fore and hind limbs of the wild type FcRγ transgenic mice that was totally missing from the animals with the ITAM mutant FcRγ (Figures 4A,B; *P* = 0.034 for FcRγ KO + WT FcRγ Tg vs. FcRγ KO + YF FcRγ Tg and *P* = 0.61 for FcRγ KO + YF FcRγ Tg vs. FcRγ KO in connection with the fore limbs; *P* = 0.012 for FcRγ KO + WT FcRγ Tg vs. FcRγ KO + YF FcRγ Tg and *P* = 0.87 for FcRγ KO + YF FcRγ Tg vs. FcRγ KO in connection with the hind limbs). However, we could not detect ankle thickness changes in the wild type FcRγ transgenic mice despite of a massive leukocyte infiltration to the joints seen on the histological sections in contrast to ITAM tyrosine mutant FcRγ expressing animals (Figures 4C,D; *P* = 0.27 for FcRγ KO + WT FcRγ Tg vs. FcRγ KO in Panel C).

### Augmentation of the Expression of the Transgenic Wild Type but not the ITAM Mutant FcRγ Enhances Arthritis Severity

**Figure 5:**
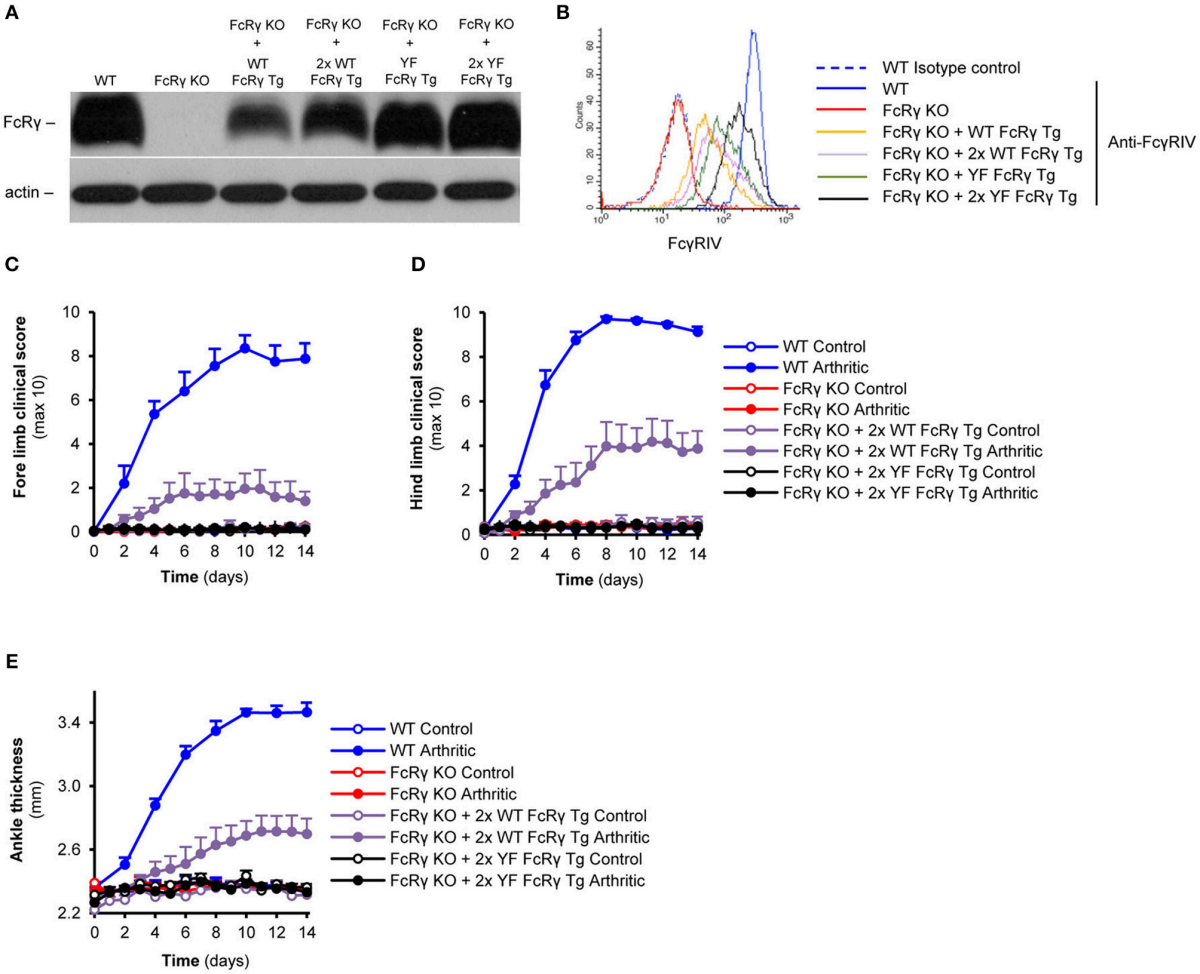
Dose-dependent effect of the wild type transgene on the severity of autoimmune arthritis in contrast to complete protection of the ITAM tyrosine mutant animals. FcRγ expression was tested by immunoblotting on whole cell lysates of neutrophils **(A)**, while cell surface expression of the FcγRIV was followed by flow cytometry **(B)**. **(C–E)** Wild type (WT), FcRγ-deficient (FcRγ KO), double wild type FcRγ transgenic (FcRγ KO + 2x WT FcRγ Tg) or double ITAM tyrosine mutant FcRγ transgenic (FcRγ KO + 2x YF FcRγ Tg) bone marrow chimeras were injected with B × N (Control) or K/B × N (Arthritic) serum intraperitonally on Day 0. Arthritis development was followed by clinical scoring of the fore limbs **(C)** and the hind limbs **(D)** and ankle thickness measurement **(E)**. The blot and the flow cytometric curves are representative of 4 independent experiments. Quantitative data show mean and SEM from 4 to 5 control and 5 to 6 arthritic serum-treated individual mice per group from 2 independent experiments **(C–E)**. See the text for actual *P* values.

For raising the expression level of the wild type and ITAM mutant transgenic FcRγ, we crossed the single transgenic (hemizygous) mice to obtain double transgenic (homozygous) animals. As shown in Figure 5A, this process resulted in an obvious enhancement in the levels of FcRγ in neutrophils of the homozygous transgenic mice compared to that of the hemizygous ones. The augmentation also appeared in the changes of the cell surface Fcγ receptor IV-expression (Figure 5B). Double transgenic wild type FcRγ bone marrow chimeras showed a robust arthritic phenotype on all limbs, while the double transgenic ITAM tyrosine mutant FcRγ chimeras were totally protected from arthritis development (Figures 5C,D; *P* = 0.04 for FcRγ KO + 2x WT FcRγ Tg vs. FcRγ KO + 2x YF FcRγ Tg and *P* = 0.70 for FcRγ KO + 2x YF FcRγ Tg vs. FcRγ KO in connection with the fore limbs; *P* = 9.2 × 10^−3^ for FcRγ KO + 2x WT FcRγ Tg vs. FcRγ KO + 2x YF FcRγ Tg and *P* = 0.96 for FcRγ KO + 2x YF FcRγ Tg vs. FcRγ KO in connection with the hind limbs). Double wild type FcRγ transgenic bone marrow chimeras also showed a significant ankle thickening compared to the homozygous ITAM mutant FcRγ transgenic bone marrow chimeras (Figure 5E; *P* = 0.01 for FcRγ KO + 2x WT FcRγ Tg vs. FcRγ KO + 2x YF FcRγ Tg and *P* = 0.96 for FcRγ KO + 2x YF FcRγ Tg vs. FcRγ KO).

Our results indicate an essential role for the FcRγ ITAM tyrosines in immune complex-triggered neutrophil cell responses and experimental arthritis.

## Discussion

Our results show that FcRγ is critical to stabilize the expression of the activating Fcγ receptors in the plasma membrane and reveals that this function is independent from the presence of its ITAM tyrosines (Figure 2). Former *in vitro* studies demonstrated the importance of a non-covalent, salt bridge-mediated interaction between the Fcγ receptor and the ITAM-containing side chain in the endoplasmic reticulum to prevent the degradation of the complex and enable the transfer of the complex to the Golgi apparatus (28). It is thought that this process is involved in the regulation of the cell surface expression of Fcγ receptors. Although our findings fit into this picture, further studies will be needed to characterize the intracellular mechanisms which stabilize and prevent the complex from degradation.

As a key observation, our structure-function studies revealed the importance of FcRγ ITAM tyrosines in immune complex-mediated neutrophil effector functions including the release of reactive oxygen species, degranulation and spreading. These findings are in accordance with previous studies of basophilic cell lines, in which the role of ITAM tyrosines was shown in FcγRIIA signaling, and in mast cells, where cell functions were abrogated after IgE-dependent stimulation (14, 16).

*In vivo* studies using murine wild type and ITAM tyrosine mutant transgenes suggested that the ITAM tyrosines are involved in degranulation, cytokine production, prostaglandin synthesis, and passive systemic anaphylaxis in mast cells (16). Even though the early *in vivo* studies on the NOTAM mouse carrying ITAM mutant FcRγ indicated the ITAM-dependence of *in vivo* cytotoxicity, uptake of immune complexes, and the cross presentation of antigens in dendritic cells (17, 18), recent papers revealed that daratumumab-induced cancer cell death (after blocking FcγRIIB) and the splenic dendritic cell-mediated activation of CD4- and CD8-positive T cells occur in an FcRγ ITAM-independent manner (19, 20). In connection to this, our *in vivo* studies indicate that FcRγ ITAM signaling is essential for mediating the initiation and progression of autoimmune arthritis. However, further studies are needed to test whether—similarly to dendritic cells—ITAM independent signaling mechanisms are also involved in other *in vivo* functions of neutrophils. It would be also informative to test the role of the ITAM tyrosines of FcRγ in other autoantibody-mediated disease models in the future to further support our findings with experimental arthritis.

*In vitro* and *in vivo* studies monitoring the phosphorylation state of ITAM tyrosines indicated that both ITAM tyrosines are phosphorylated upon stimulation, but the carboxy-terminal tends to be dephosphorylated much faster in mast cells (15). However, it is not known how the phosphorylation state of the two tyrosines is regulated in neutrophils.

It should be noted that not only FcγRIII and FcγRIV—the receptor that is alone sufficient to induce arthritis (7)—, but other receptors also are associated with FcRγ, including Paired Immunglobulin-like receptor-A (PIR-A), Leukocyte immunglobulin-like receptor 1C (LILR1C), Osteoclast-associated receptor (OSCAR), T cell-interacting, activating receptor on myeloid cells-1 (TARM1), Dectin-2 or some β integrins in neutrophils (29–36). Further studies are needed whether the ligand binding or crosslinking of these receptors require the function of the FcRγ ITAM tyrosines to enable the expression and induction of cellular responses.

As shown in Figures 2 and 5, the expression levels of the wild type and the FcRγ ITAM mutant transgenic neutrophils are different. The presence of the complex between the ITAM-containing molecule and the Fcγ receptor has been reported in the endoplasmic reticulum, which is likely the cellular organism where the stability of the complex and the degradation of the components is actively regulated that may require the presence of the ITAM tyrosines (28). It is also possible that the copy numbers of the two transgenes are different. A third possibility is that different enhancer and silencer elements are involved in the regulation of the expression of the two transgenes as they may not be present at the same place in the genome. Most importantly, despite of the higher level, ITAM mutant FcRγ was not able to restore neutrophil effector responses and reverse the development of autoimmune arthritis, which further support our conclusions.

Compared to the hemizygous animals, the double transgenic wild type FcRγ mice could reverse more effectively the development of autoimmune arthritis on the FcRγ-deficient background, indicating that the disease progression is highly dependent on the level of the signaling molecule. This can be the explanation of why the ankle thickness was not significantly changed, while the increase in the more sensitive clinical score was significant and the immune cell infiltration was massive and robust in the single wild type FcRγ ITAM transgenic animals.

As the absence of FcRγ was not required for neutrophil migration in experimental arthritis, it can be hypothesized that FcRγ ITAM tyrosines are also not involved in the accumulation of neutrophils at the site of inflammation (37). These tyrosines could rather have a role in mediating immune complex-triggered responses at the site of tissue damage as we showed it in connection with several other molecules involved in neutrophil Fcγ receptor signaling (21, 24, 26).

Besides neutrophils, other cell types like macrophages, mast cells or platelets have been indicated in the pathogenesis of experimental arthritis (38–41). It would be interesting to test the role of the ITAM tyrosines of FcRγ in these cell populations in joint inflammation. Collectively, our studies revealed the critical role of FcRγ ITAM tyrosines in immune complex-mediated activation of neutrophils and the development and progression of autoantibody-induced autoimmune arthritis. Importantly, our studies provide the first direct *in vivo* evidence for the role of FcRγ ITAM tyrosines in a neutrophil-dependent *in vivo* arthritis model. Understanding these molecular mechanisms can serve as new therapeutic targets in the treatment of some autoimmune diseases in the future (e.g., by the development of molecules which may mask the ITAM tyrosines of FcRγ).

## Author Contributions

TN, AM, and ZJ designed the work, interpreted the results and wrote the paper. TN, KF, MS, PA, and ZJ performed the experiments and analyzed the data. TS developed and provided the wild type and ITAM mutant FcRγ transgenic mouse strains.

### Conflict of Interest Statement

The authors declare that the research was conducted in the absence of any commercial or financial relationships that could be construed as a potential conflict of interest.

## Abbreviations

FcRγ: Fc receptor γ-chain
IC: Immune complex
ITAM: Immunoreceptor tyrosine-based activation motif
FcR: Fc receptor
GPI: Glycosylphosphatidylinositol
PCR: Polymerase chain reaction
PMA: Phorbol myristate acetate
WT: Wild type.

## References

[1] Bruhns P Jonsson F. Mouse and human FcR effector functions. Immunol Rev. (2015) 268:25–51. 10.1111/imr.12350 26497511

[2] Nimmerjahn F. Activating and inhibitory FcγRs in autoimmune disorders. Springer Semin Immunopathol. (2006) 28:305–19. 10.1007/s00281-006-0052-1 17115158

[3] Futosi K Mócsai A. Tyrosine kinase signaling pathways in neutrophils. Immunol Rev. (2016) 273:121–39. 10.1111/imr.12455 27558332

[4] Ludwig RJ Vanhoorelbeke K Leypoldt F Kaya Z Bieber K McLachlan SM. Mechanisms of autoantibody-induced pathology. Front Immunol. (2017) 8:603. 10.3389/fimmu.2017.00603 28620373 PMC5449453

[5] Bruhns P. Properties of mouse and human IgG receptors and their contribution to disease models. Blood (2012) 119:5640–9. 10.1182/blood-2012-01-380121 22535666

[6] Jakus Z Németh T Verbeek JS Mócsai A. Critical but overlapping role of FcγRIII and FcγRIV in activation of murine neutrophils by immobilized immune complexes. J Immunol. (2008) 180:618–29. 10.4049/jimmunol.180.1.618 18097064 PMC2647079

[7] Mancardi DA Jonsson F Iannascoli B Khun H Van Rooijen N Huerre M. Cutting edge: the murine high-affinity IgG receptor Fcγriv is sufficient for autoantibody-induced arthritis. J Immunol. (2011) 186:1899–903. 10.4049/jimmunol.1003642 21248252

[8] Hobday PM Auger JL Schuneman GR Haasken S Verbeek JS Binstadt BA. Fcγ receptor III and Fcγ receptor IV on macrophages drive autoimmune valvular carditis in mice. Arthritis Rheumatol. (2014) 66:852–62. 10.1002/art.38311 24757138 PMC4012861

[9] van Vugt MJ Heijnen IA Capel PJ Park SY Ra C Saito T FcR γ-chain is essential for both surface expression and function of human FcγRI (CD64) *in vivo*. Blood (1996) 87:3593–9. 8611682

[10] Takai T Li M Sylvestre D Clynes R Ravetch JV. FcR γ chain deletion results in pleiotrophic effector cell defects. Cell (1994) 76:519–29. 8313472 10.1016/0092-8674(94)90115-5

[11] Nimmerjahn F Bruhns P Horiuchi K Ravetch JV. FcγRIV: a novel FcR with distinct IgG subclass specificity. Immunity (2005) 23:41–51. 10.1016/j.immuni.2005.05.010 16039578

[12] Ji H Ohmura K Mahmood U Lee DM Hofhuis FMA Boackle SA. Arthritis critically dependent on innate immune system players. Immunity (2002) 16:157–68. 10.1016/S1074-7613(02)00275-3 11869678

[13] Corr M Crain B. The role of FcγR signaling in the K/B x N serum transfer model of arthritis. J Immunol. (2002) 169:6604–9. 10.4049/jimmunol.169.11.6604 12444173

[14] Daniels AB Worth RG Dickstein RJ Dickstein JS Kim-Han TH Kim MK. Analysis of FcγRIIA cytoplasmic tail requirements in signaling for serotonin secretion: evidence for an ITAM-dependent, PI3K-dependent pathway. Scand J Immunol. (2010) 71:232–9. 10.1111/j.1365-3083.2010.02369.x 20384866 PMC2855141

[15] Yamashita T Suzuki R Backlund PS Yamashita Y Yergey AL Rivera J. Differential dephosphorylation of the FcRγ immunoreceptor tyrosine-based activation motif tyrosines with dissimilar potential for activating Syk. J Biol Chem. (2008) 283:28584–94. 10.1074/jbc.M802679200 18715866 PMC2568911

[16] Sakurai D Yamasaki S Arase K Park SY Arase H Konno A. FcεRIγ-ITAM is differentially required for mast cell function *in vivo*. J Immunol. (2004) 172:2374–81. 10.4049/jimmunol.172.4.2374 14764707

[17] de Haij S Jansen JH Boross P Beurskens FJ Bakema JE Bos DL. *In vivo* cytotoxicity of type I CD20 antibodies critically depends on Fc receptor ITAM signaling. Cancer Res. (2010) 70:3209–17. 10.1158/0008-5472.CAN-09-4109 20354182

[18] Boross P van Montfoort N Stapels DA van der Poel CE Bertens C Meeldijk J. FcRγ-chain ITAM signaling is critically required for cross-presentation of soluble antibody-antigen complexes by dendritic cells. J Immunol. (2014) 193:5506–14. 10.4049/jimmunol.1302012 25355925

[19] Overdijk MB Jansen JH Nederend M Lammerts van Bueren JJ Groen RW Parren PW. The Therapeutic CD38 monoclonal antibody daratumumab induces programmed cell death via Fcγ receptor-mediated cross-linking. J Immunol. (2016) 197:807–13. 10.4049/jimmunol.1501351 27316683

[20] Lehmann CHK Baranska A Heidkamp GF Heger L Neubert K Luhr JJ. DC subset-specific induction of T cell responses upon antigen uptake via Fcγ receptors *in vivo*. J Exp Med. (2017) 214:1509–28. 10.1084/jem.20160951 28389502 PMC5413326

[21] Kovács M Németh T Jakus Z Sitaru C Simon E Futosi K. The Src family kinases Hck, Fgr, and Lyn are critical for the generation of the *in vivo* inflammatory environment without a direct role in leukocyte recruitment. J Exp Med. (2014) 211:1993–2011. 10.1084/jem.20132496 25225462 PMC4172222

[22] Németh T Futosi K Szilveszter K Vilinovszki O Kiss-Papai L Mócsai A. Lineage-specific analysis of Syk function in autoantibody-induced arthritis. Front Immunol. (2018) 9:555. 10.3389/fimmu.2018.00555 29616043 PMC5867294

[23] Kouskoff V Korganow AS Duchatelle V Degott C Benoist C Mathis D. Organ-specific disease provoked by systemic autoimmunity. Cell (1996) 87:811–22. 8945509 10.1016/s0092-8674(00)81989-3

[24] Jakus Z Simon E Frommhold D Sperandio M Mócsai A. Critical role of phospholipase Cγ2 in integrin and Fc receptor-mediated neutrophil functions and the effector phase of autoimmune arthritis. J Exp Med. (2009) 206:577–93. 10.1084/jem.20081859 19273622 PMC2699137

[25] Németh T Futosi K Hably C Brouns MR Jakob SM Kovács M. Neutrophil functions and autoimmune arthritis in the absence of p190RhoGAP: generation and analysis of a novel null mutation in mice. J Immunol. (2010) 185:3064–75. 10.4049/jimmunol.0904163 20675588 PMC3064944

[26] Németh T Futosi K Sitaru C Ruland J Mócsai A. Neutrophil-specific deletion of the CARD9 gene expression regulator suppresses autoantibody-induced inflammation *in vivo*. Nat Commun. (2016) 7:11004. 10.1038/ncomms11004 27032818 PMC4821996

[27] Mócsai A Zhang H Jakus Z Kitaura J Kawakami T Lowell CA. G-protein-coupled receptor signaling in Syk-deficient neutrophils and mast cells. Blood (2003) 101:4155–63. 10.1182/blood-2002-07-2346 12531806

[28] Kurosaki T Gander I Ravetch JV. A subunit common to an IgG Fc receptor and the T-cell receptor mediates assembly through different interactions. Proc Natl Acad Sci USA. (1991) 88:3837–41. 1827205 10.1073/pnas.88.9.3837 PMC51548

[29] Kubagawa H Burrows PD Cooper MD. A novel pair of immunoglobulin-like receptors expressed by B cells and myeloid cells. Proc Natl Acad Sci USA. (1997) 94:5261–6. 9144225 10.1073/pnas.94.10.5261 PMC24666

[30] Maeda A Kurosaki M Kurosaki T. Paired immunoglobulin-like receptor (PIR)-A is involved in activating mast cells through its association with Fc receptor γ chain. J Exp Med. (1998) 188:991–5. 9730901 10.1084/jem.188.5.991 PMC2213385

[31] Hoelsbrekken SE Fossum S Dissen E. Molecular cloning of LILRC1 and LILRC2 in the mouse and the rat, two novel immunoglobulin-like receptors encoded by the leukocyte receptor gene complex. Immunogenetics (2005) 57:479–86. 10.1007/s00251-005-0014-0 16041585

[32] Merck E Gaillard C Scuiller M Scapini P Cassatella MA Trinchieri G. Ligation of the FcRγ chain-associated human osteoclast-associated receptor enhances the proinflammatory responses of human monocytes and neutrophils. J Immunol. (2006) 176:3149–56. 10.4049/jimmunol.176.5.3149 16493074

[33] Radjabova V Mastroeni P Skjodt K Zaccone P de Bono B Goodall JC. TARM1 Is a novel leukocyte receptor complex-encoded ITAM receptor that costimulates proinflammatory cytokine secretion by macrophages and neutrophils. J Immunol. (2015) 195:3149–59. 10.4049/jimmunol.1401847 26311901 PMC4595996

[34] Kerscher B Willment JA Brown GD. The Dectin-2 family of C-type lectin-like receptors: an update. Int Immunol. (2013) 25:271–7. 10.1093/intimm/dxt006 23606632 PMC3631001

[35] Jakus Z Fodor S Abram CL Lowell CA Mócsai A Immunoreceptor-like signaling by β2 and β3 integrins. Trends Cell Biol. (2007) 17:493–501. 10.1016/j.tcb.2007.09.001 17913496

[36] Fodor S Jakus Z Mócsai A. ITAM-based signaling beyond the adaptive immune response. Immunol Lett. (2006) 104:29–37. 10.1016/j.imlet.2005.11.001 16332394

[37] Monach PA Nigrovic PA Chen M Hock H Lee DM Benoist C. Neutrophils in a mouse model of autoantibody-mediated arthritis: critical producers of Fc receptor γ, the receptor for C5a, and lymphocyte function-associated antigen 1. Arthritis Rheum. (2010) 62:753–64. 10.1002/art.27238 20191628 PMC3057458

[38] Solomon S Rajasekaran N Jeisy-Walder E Snapper SB Illges H. A crucial role for macrophages in the pathology of K/B x N serum-induced arthritis. Eur J Immunol. (2005) 35:3064–73. 10.1002/eji.200526167 16180250

[39] Lee DM Friend DS Gurish MF Benoist C Mathis D Brenner MB. Mast cells: a cellular link between autoantibodies and inflammatory arthritis. Science (2002) 297:1689–92. 10.1126/science.1073176 12215644

[40] Nigrovic PA Binstadt BA Monach PA Johnsen A Gurish M Iwakura Y. Mast cells contribute to initiation of autoantibody-mediated arthritis via IL-1. Proc Natl Acad Sci USA. (2007) 104:2325–30. 10.1073/pnas.0610852103 17277081 PMC1892913

[41] Boilard E Nigrovic PA Larabee K Watts GF Coblyn JS Weinblatt ME. Platelets amplify inflammation in arthritis via collagen-dependent microparticle production. Science (2010) 327:580–3. 10.1126/science.1181928 20110505 PMC2927861

